# Predictable Performance for QoS-Sensitive, Scalable, Multi-tenant Function-as-a-Service Deployments

**DOI:** 10.1007/978-3-030-58858-8_14

**Published:** 2020-08-18

**Authors:** Andrzej Kuriata, Ramesh G. Illikkal

**Affiliations:** 6 IT University of Copenhagen, Copenhagen, Denmark; 7 University of British Columbia, Vancouver, BC Canada; 8 Intel Technology Poland, Gdansk, Poland; 9 Intel Corp., Santa Clara, CA USA

**Keywords:** Performance, Telemetry, Scheduling

## Abstract

In this paper we present the results of our studies focused on enabling predictable performance for functions executing in scalable, multi-tenant Function-as-a-Service environments. We start by analyzing QoS and performance requirements and use cases from the point of view of End-Users, Developers and Infrastructure Owners. Then we take a closer look at functions’ resource utilization patterns and investigate functions’ sensitivity to those resources. We specifically focus on the CPU microarchitecture resources as they have significant impact on functions’ overall performance. As part of our studies we have conducted experiments to research the effect of co-locating different functions on the compute nodes. We discuss the results and provide an overview of how we have further modified the scheduling logic of our containers orchestrator (Kubernetes), and how that impacted functions’ execution times and performance variation. We have specifically leveraged the low-level telemetry data, mostly exposed by the Intel® Resource Director Technology (Intel® RDT) [1]. Finally, we provide an overview of our future studies, which will be centered around node-level resource allocations, further improving a function’s performance, and conclude with key takeaways.

## Introduction

The general Cloud Computing model relies on centralizing computing power and then re-distribution of this computing power among multiple users and tenants. The benefits of such approach, among others, are inherit scalability and, from the end user perspective, simplified resources management.

Additional layers built on top of Cloud Computing, like Function-as-a-Service deployments, release the burden of managing hardware and software resources, from service developers, even further. At the same time, however, resource providers must ensure that performance of services is stable and independent from performance and resource utilization of other services running at the same time on the same set of resources.

In this paper we investigate the methods for improving services’ performance stability, which we view as an important aspect of overall Quality of Service.

### The Importance of Predictable Functions Performance

The predictability of function execution performance (most often function execution time) is important from several reasons. Here are the QoS and performance expectations from Users, Developers/Application Owners and Infrastructure Owners/Admins:

1. End users value performance consistency for practical reasons. Unexpected application slowdown can cause frustration but also can negatively impact important business operations. Positive overall user experience requires assurance that service response time will have low, predictable latency.
2. Developers/Application Owners want predictable billing. Most often Infrastructure Owners charge by millisecond of function execution time. Any churn in function execution time can impact billing negatively. The reason for inconsistent function execution time is only partially in control of Developers/Application Owners (e.g. associated with function logic processing the input). Other issues, like resource contention or noisy neighbor problems in shared-resources, multi-tenant environments, can be solved only by the Infrastructure Owners.
3. Infrastructure Owners want to provide predictable performance for their Users and at the same time maximize resources utilization as this improves their Total Cost of Ownership. As for FaaS, many CSPs adopt sub second billing, which puts stringent SLA requirements in terms of run-to-run variability. When Infrastructure Owners have awareness which resources are most critical for stable functions’ performance, they can better optimize their scheduling policies to optimize their computing resources utilization.

In this study we define predictable performance in relation to Coefficient of Variation (CV) for function execution time. The CV itself is defined as [2]:

1 $$ c_{v} = \frac{\sigma }{\mu } $$

Where, $c_{v}$ is a coefficient of variation, $\sigma$ is a standard deviation and $\mu$ is a mean.

We consider function to have predictable performance when its CV is less than or equal to 15%. Otherwise, we consider the performance to be unpredictable. When average function execution time is 1 s, and resource utilization billing is done at 100 ms granularity, then 15% execution time churn corresponds to up to 2 billing cycles, which we consider tolerable from function owner perspective.

**Fig. 1.**
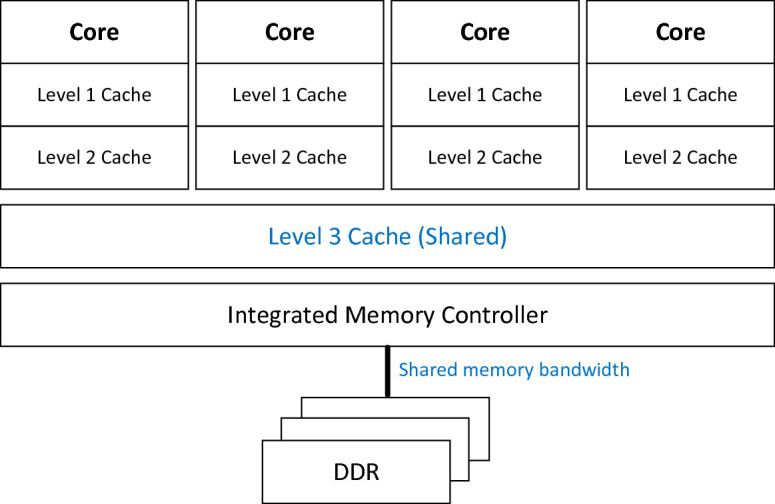
High level view of Intel Xeon processor

FaaS deployments are intrinsically multi-tenant and expected to scale rapidly, on-demand. To enable such scaling, without sacrificing performance, we propose to pay special attention to CPU microarchitecture resources utilization, as it directly correlates with functions’ performance. Here is the high-level view of resources for Intel Xeon Processor (Fig. 1).

Especially shared resources, like memory bandwidth to DRAM (controlled by the Integrated Memory Controller) and Last Level Cache (i.e. Third Level Cache) should be closely monitored, as minimizing contention on those resources improves overall functions’ performance. Also, for multi-socket platforms, crossing socket or NUMA node boundary might be associated with performance penalty (due to narrower remote memory bandwidth). The study analyzing impact of memory latency and memory bandwidth to the workload’s performance is described in [3].

In general, the pool of CPU cores is also a constrained resource on which contention might happen. But we leave the task of allocating software threads to CPU cores to the Linux scheduler and did not interfere with that in our study.

## Analyzing Functions Performance and Performance Predictability

In the following sections we describe how we were analyzing functions performance. We have started with gathering information about functions characteristics, especially resources sensitivity patterns. That enabled us to further analyze performance related problems and propose solutions.

### Test Stack and Test Functions

We’ve conducted our experiments on a 4-node Kubernetes cluster, with 1 master and 3 worker nodes. All nodes are 2-socket Intel Skylake platforms (Intel® Xeon® Gold 6140 CPU @ 2.30 GHz, with 18 physical cores and HyperThreading enabled).

For the software stack we used: Kubernetes as containers orchestrator, Docker as containers engine/runtime, OpenFaaS as a FaaS framework and Intel Workload Collocation Agent [4] as a main telemetry framework.

In our experiments we are using following functions:

- Incept, which uses Tensorflow for image recognition
- Nmt, which uses Tensorflow for English to German translation
- Sgemm, which does single precision floating General Matrix Multiply
- Stream, the STREAM benchmark [5].

### Introduction to Top-Down Microarchitecture Analysis Methodology

Our test functions have been profiled using Top-Down Microarchitecture Analysis methodology [6, 7]. This approach facilitates finding categories of platform resources, and individual resources, that are most critical to the workload (e.g. function) and can limit performance when not available. The results of the profiling, at high CPU utilization (ranging from 95 to 100%), are presented in the Table 1 below.

**Table 1.** TMA profiles of the test functions

Function	A	B	C	D	E	F	G
Incept	1.13	60.7	17.0	56.1	22.2	4.7	3.1
Nmt	2.35	52.4	22.8	60.6	10.8	5.8	0.1
Sgemm	0.49	32.8	17.7	25.8	40.8	15.6	29.4
Stream	2.40	71.6	1.5	93.0	5.5	0.1	0.1

Where: A – Last Level Cache Misses per 1000 Instructions, B – Memory Bandwidth Utilization [%], C – Frontend Bound [%], D – Backend Bound [%], E – Retiring [%], F – Bad Speculation, G – Flops Used/Flops Max [%].

This knowledge can be leveraged in optimizing scheduling and load balancing logic, so that functions’ performance is not hampered by the lack of critical platform resources. This is specifically important in large scale, multi-tenant deployment where noisy neighbor effects are most prominent.

### Platform Resources Utilization Monitoring

During functions’ execution we collect telemetry data to better understand resources utilization patterns. For each function instance, per each call, we are collecting the following:

- Memory bandwidth utilization – exposed by the Linux ‘resctrl’ filesystem, the source of data is Intel RDT Memory Bandwidth Monitoring technology
- Last Level Cache Occupancy – exposed by the Linux ‘resctrl’ filesystem, the source of data is Intel RDT Cache Monitoring Technology
- Last Level Cache Misses Per Kilo Instructions – exposed by the platform as a CPU architectural performance monitoring event, can be collected, for example: via Linux perf tool
- CPU utilization – exposed by the Linux CGroup filesystem

We also record function execution times as an indicator of a function’s performance.

Having insight into nodes’ resource utilization and availability is critical in order to improve placement of functions on the nodes. Here are the most important telemetry data that we collect per each compute node:

- CPU utilization – exposed by the Linux/proc/stat file
- Memory bandwidth utilization – exposed by the CPU Performance Monitoring Unit (of Integrated Memory Controller), can be calculated from events collected, for example via Linux perf tool
- Average memory latency – exposed by the CPU Performance Monitoring Unit (of Integrated Memory Controller), can be calculated from events collected, for example via Linux perf tool.

## Improving Performance Predictability

### Analyzing Functions’ Co-location Cases

In this experiment we use “hey” [8] to stress the test functions. We start from light load (low Request-Per-Second values) and continue stressing functions up to the point where all cores (36 total for 2 sockets, 18 cores per function) on the platform are utilized, thus translating to high RPS values. Theoretically, functions with moderate memory bandwidth consumption should co-exist better on the same node than functions with high memory bandwidth requirements. The reason is less contention on the resource required by both functions. We should also see improved function execution times and lower resources utilization when functions are not competing over the same, shared resource.

**Fig. 2.**
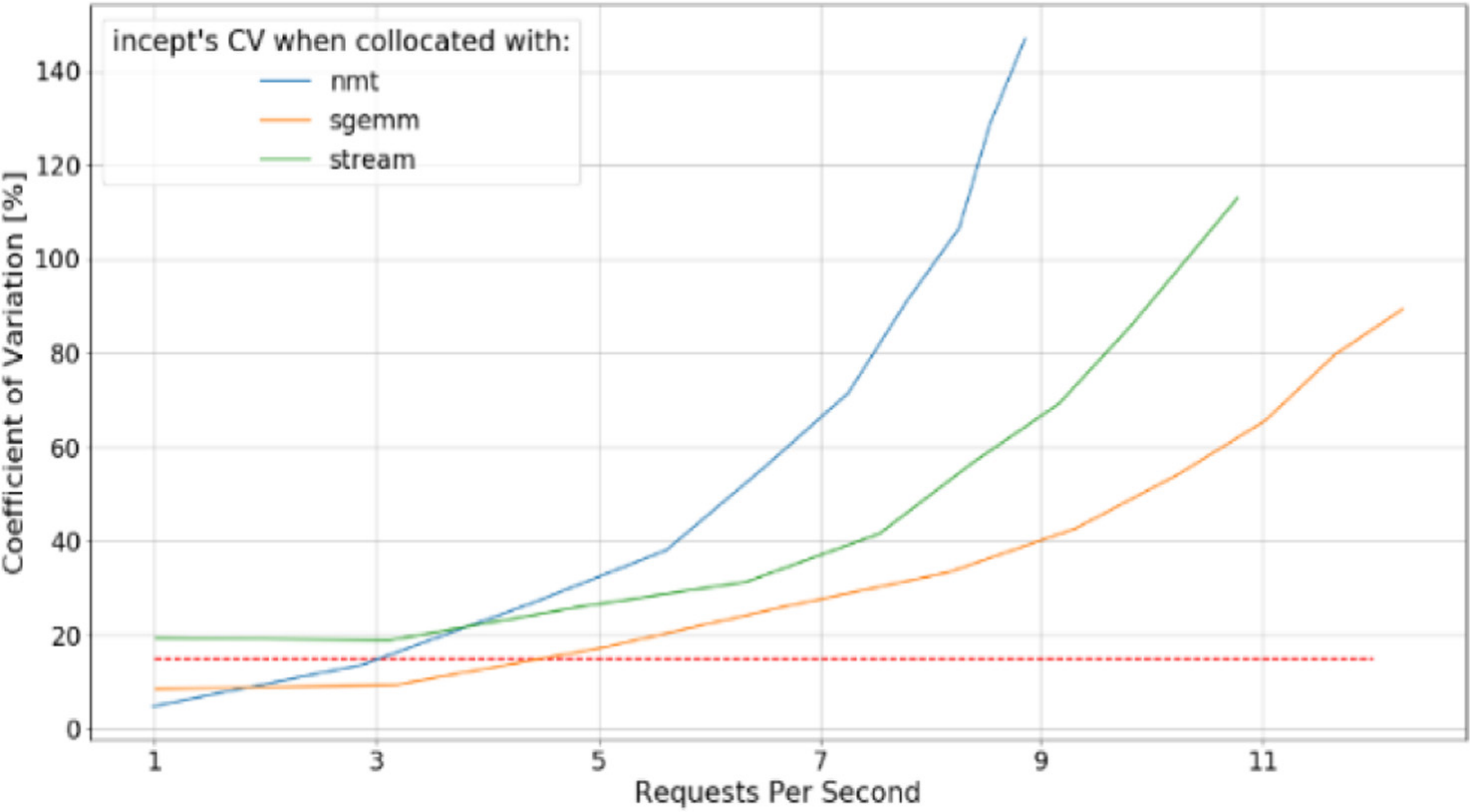
Incept’s CV when scheduled with other test functions (red, dashed line at 15% represents our threshold, below which, we consider function to have predictable performance) (Color figure online)

The results for the “Incept” function scheduled along with other functions are depicted below (Fig. 2):

We can observe that, if Incept is located with Sgemm it achieves the best performance predictability (lowest CV values across the RPS range) and best throughput (lowest average function execution time). An optimal scheduler should co-locate Incept with Sgemm, rather than Nmt or Stream. The worst colocation case is placing Incept and Nmt on the same node, and optimal scheduler should avoid that. Incept and Nmt are poor candidates for colocation because they are heavy memory bandwidth users and natural contenders for this resource.

The table below presents comparison of average node resources utilization when Incept is collocated with Nmt (sub-optimal placement) and when Incept is collocated with Sgemm (optimal placement) in case when all CPU cores on the platform are utilized (Table 2).

**Table 2.** Comparison of average node resources utilization between optimal (Incept + Sgemm) and sub-optimal (Incept + Nmt) co-location scenarios

Resource	Optimal placement	Sub-optimal placement
Memory bandwidth utilization [%]	30	47
Average memory latency [ns]	41	92
CPU Utilization [%]	55	78
LLC MPKI^a^	50	110

^a^LLC MPKI – Last Level Cache Misses Per Kilo Instructions

Sub-optimal placement results in almost 20% higher memory bandwidth utilization, increased memory latency, and around 20% higher CPU utilization. And as we’ve seen before, wrong placement decision ultimately impacts function execution time and execution time variability.

### Scheduling Improvements

By leveraging per-container telemetry (especially memory bandwidth utilization) and per-node resource availability we tried to improve the scheduling logic. In Kubernetes, which we are using as our containers’ orchestrator, scheduling is a two-stage process. In the first step (filtering) we exclude any nodes without enough available memory bandwidth. In the second step (prioritization) we assign scores to the nodes and select the node with the highest score. Here are the scoring categories:

- Available memory bandwidth – nodes are sorted with available memory bandwidth in descending order. The node with maximum available memory bandwidth is assigned highest score, and the one with the lowest amount of available memory bandwidth is assigned the lowest score.
- Memory Latency – nodes are sorted and assigned scored based on the memory latency (lower values are preferred over higher values)
- CPU utilization – nodes are sorted based on available CPU (more available CPU equals to higher score)

Scores from all categories are summarized per node and the node with highest overall score is selected.

**Fig. 3.**
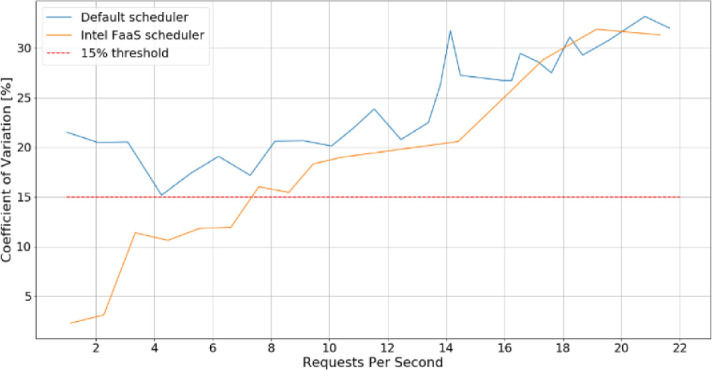
The comparison of Incept’s CV when using default scheduler vs. scheduler which is memory bandwidth and memory latency aware.

Graph below present comparison of Incept’s CV when using default scheduling logic vs scheduling logic which takes memory bandwidth and memory latency into account. Scheduling enhancement were done by leveraging Kubernetes scheduler extender mechanism [9] (Fig. 3).

For lower RPS (up to around 7), the scheduler extender reduces CV to acceptable level (15%). Execution time also improves slightly, which can result in improved cluster throughput. Those results can be further improved with RDT Memory Bandwidth Allocation feature, which we plan to leverage in future experiments.

## Future Work

As a next step we plan to research how at-node-level allocation of resources (e.g. by using Intel RDT Memory Bandwidth Allocation and Cache Allocation Technology) impacts functions’ performance.

We would also like to deepen studies on differentiated performance for QoS-sensitive workloads. The Service Level Agreements are commonly used for managing QoS. At its simplest form the SLA can be expressed as a two-level function prioritization agreement, distinguishing between high and low priority tasks (e.g. functions). We’d like to research how high-level SLAs can be mapped to resource allocations and how allocations enforcement can be used for improving performance predictability even further.

## Conclusions

We have demonstrated that low level telemetry data (especially related to memory bandwidth utilization) can be used to improve functions performance predictability, for example by optimizing scheduling logic.

By leveraging memory bandwidth monitoring capabilities of Intel Resource Director Technology, we were able to optimize resource utilization and provide best performance for memory bandwidth sensitive workloads (ML-based inference workloads in our experiment).
